# Neighborhood Walkability Is Not Associated with Adults’ Sedentary Behavior in the Residential Setting: Evidence from Breda, The Netherlands

**DOI:** 10.3390/ijerph16183487

**Published:** 2019-09-19

**Authors:** Menno Luijkx, Marco Helbich

**Affiliations:** Department of Human Geography and Spatial Planning, Faculty of Geosciences, Utrecht University, Princetonlaan 8a, 3584 CB Utrecht, The Netherlands; m.luijkx@students.uu.nl

**Keywords:** sedentary behaviors, neighborhood walkability, adults

## Abstract

Sedentary behavior has negative health effects. It is assumed that the walkability of the living environment is related to the amount of time spent on sedentary behavior in the residential setting. However, evidence on such a relation is still scarce, and results are contradictory. Therefore, we examined to what extent residential neighborhood walkability is associated with a variety of sedentary behaviors that frequently occur in the residential setting among adults. We carried out a cross-sectional survey using the domain-specific sedentary behavior questionnaire among adults in Breda, the Netherlands. Respondents’ walkability of the living environment was assessed objectively by means of road network buffers. We employed gamma generalized linear regressions to assess correlations between multiple sedentary behaviors and neighborhood walkability. We found no significant associations between residential neighborhood walkability and sedentary behavior levels. The lack of correlations was consistent across buffer sizes. Our models showed that adults with a higher education, a job, and a driver’s license spent significantly less time on sedentary behaviors. Our findings suggest that person-level characteristics should be targeted when developing intervention strategies to counteract sedentary time, rather than intervening in the walkability of the residential living environment.

## 1. Introduction

There is growing evidence that pronounced levels of sedentary behaviors have negative health effects [1,2]. Sedentary behavior refers to behavior with a low energy expenditure (with ≤1.5 metabolic equivalents) while being in a sitting or reclining position [3]. Studies showed positive associations between adults who spent greater time on sedentary behavior and an increased risk of several diseases (e.g., obesity, cardiovascular diseases) [1]. As these findings were independent of people’s physical activity levels, sedentary behavior cannot be seen as synonymous with physical inactivity [4]. Adults who meet recommended physical activity guidelines (e.g., ≥150 min of moderate intensity physical activity per week) still compromise their health by being sedentary [5].

Technological innovations have given rise to increasingly physically passive lifestyles in which more consecutive hours are spent on sedentary behavior (e.g., watching television, playing computer games) [3]. Approximately 20% of European adults spend about 7.5 h per day sitting down, which illustrates the high prevalence of sedentary behavior [6,7]. The figure for the Netherlands, namely 32%, is significantly higher than the European average (19%) [6]. Meta-analysis indicated an increase in adults’ all-cause mortality with 6 to 8 h of sedentary time accumulated over a day [8].

There has been a paradigm shift in studies on health behaviors. At first, studies focused predominantly on individual-level determinants, including people’s motivation, demographics, socioeconomic characteristics, family situation, etc. [9]. Nowadays, ecological models are utilized to better understand the complexity of people’s sedentary behavior. An ecological model considers interdependencies between personal and interpersonal characteristics, as well as the physical and social environment as health-influencing factors [10]. Here, the built environment is understood as all buildings, public spaces, and objects in one’s surroundings that are human-made or modifiable [11,12]. Attributes of the built environment influence physical activity [13] by presenting cues to individuals (e.g., pavements) that prompt behavioral responses in return (e.g., walking) [14]. This makes it likely that the built environment may also be associated with sedentary behavior [15]. Environments that encourage individuals to spend more time on active behavior (e.g., walking, cycling, playing sports) are theoretically expected to decrease the amount of time that individuals spend sedentarily [16,17].

One way to capture the complexity of the built environment is to quantify the walkability of neighborhoods. Such indices capture the extent to which a built environmental setting supports the act of walking derived through a geographical information system (GIS) [18]. Although there is no universal operationalization, walkability indices usually consider a neighborhood’s land-use mix (i.e., the diversity of land uses), street connectivity (i.e., the directness of streets and the availability of alternative routes), and residential density (i.e., the number of residents) [19]. 

For example, prior studies on walkability found that adults living in highly walkable neighborhoods walk significantly more than their counterparts in low-walkable neighborhoods [20]. This is primarily true for walking for transport [21], while findings for recreational walking are less clear [22]. Others found no link between residential density and the total amount of walking [23]. Nevertheless, adults in highly walkable neighborhoods spend more time on active behavior, which is why neighborhood walkability might also play a significant role concerning people’s sedentary behavior. However, to date, only a few studies have reported on such a relationship [24,25,26,27,28]. Some of these studies showed that adults living in highly walkable neighborhoods spend less time watching television [26,27], while others reported that less time is spent sitting in cars [25,26]. However, yet other studies found that living in highly walkable neighborhoods results in more overall sitting time [28], or no significant association with television viewing time was found [24]. 

In addition to these contradictory findings, existing evidence is limited in two important ways. First, there is a tendency to focus either on a specific sedentary behavior or on a total sedentary time component [29]. Second, from a methodological point of view, studies measured neighborhood walkability on a postcode or census tract level. These fictive areas do not necessarily reflect people’s actual living environment [30], and may lead to an overestimation or underestimation of the sedentary behavior–walkability correlation. 

In light of these limitations, the present study examined potential correlations between residential neighborhood walkability and a variety of sedentary behaviors that occur frequently in the residential setting among adults. Our leading research question was: To what extent does neighborhood walkability influence time spent on different sedentary behaviors in adults? It was hypothesized that adults living in highly walkable neighborhoods spend less time on sedentary behavior than those in less walkable neighborhoods. 

## 2. Materials and Methods 

### 2.1. Study Design and Study Population 

This cross-sectional study was conducted in the city of Breda (184,000 inhabitants), the Netherlands. Breda recently merged with several smaller municipalities, which has led to a contrasting built environment landscape. The historic inner city is relatively dense and has a rich land-use mix of residential, retail, and leisure areas, while the outer parts are formerly independent municipalities, and are more homogeneous in terms of land use. Therefore, it was an appropriate study area for our investigation. 

A total of 1900 households randomly drawn from the Dutch register of addresses and buildings (Basisregistratie Adressen en Gebouwen (BAG), 2019) were sent a letter between May and June 2019 inviting them to participate in the study. The eligibility criteria for household members were 18 years or older, able to walk without assistance, and capable of reading Dutch. After a low response rate (7%), the survey was promoted through social media channels (i.e., Facebook, LinkedIn) related to the municipality of Breda. This strategy increased the response rate to 11%. Twenty of the 205 recruited participants were excluded from analysis because their residential location was outside Breda. Another two participants were excluded because they exceeded 24 h of sedentary behavior per day either during week or at weekends. Therefore, the final sample consisted of 183 participants.

### 2.2. Outcomes

The well-tested domain-specific sedentary time questionnaire (SIT-Q-7d) was used to measure the amount of time the participants spent on sedentary behaviors. The questions are about the amount of time spent sitting or lying down in the previous seven days [29]. The participants were asked to recall 10 sedentary behaviors (Table 1) for weekdays and weekend days separately. This questionnaire was preferred over more objective ones, because it captures sedentary behaviors in specific contexts (e.g., watching television, reading, listening to music) [31]. Further, given our objective, we were interested in the sedentary behaviors that occur most frequently in the participants’ living environment.

The response categories differed per class and were organized into either 15-min, 30-min, or hourly blocks, with the largest category being ≥8 h. The SIT-Q-7d scoring system assigns midpoint values to each response category [32]. For instance, the midpoint for the response category ‘4 to 6 h’ is 5 h. The midpoints for the open-ended response categories (e.g., ‘more than 6 h’) were calculated by adding half of the difference between the upper and the lower cut-point of the previous response category [32]. The previous response category for ‘more than 6 h’ was ‘4 to 6 h’, meaning that a midpoint value of 7 h was assigned. We calculated the means for the four classes as follows [28]: (average weekday sedentary behavior × 5) + (average weekend day sedentary behavior × 2)/7.

### 2.3. Walkability Index 

Neighborhood walkability was assessed objectively by means of a GIS-based walkability index that is based on land-use mix, residential density, and street connectivity. Each index component was calculated for road network buffers centered on the respondent’s home. Road network buffers were found to be more accurate and to correlate more strongly with health behaviors than circular ones [33]. Our buffer conceptualization used a detailed buffer with a 50-m trim [33] and three distances (i.e., 200 m, 400 m, and 800 m). The 200-m buffer was intended to capture the immediate neighborhood context. The larger ones refer to acceptable walking distances to local destinations [34]. 

Land-use mix represents the heterogeneity of land uses (e.g., residential, shopping) within a buffer. Diverse land uses typically offer more non-residential destinations, supporting walking trips [35]. To assess land-use diversity, we extracted each building’s usage, and recoded them into five mutually exclusive categories (i.e., residential, commercial, institutional, industrial, and recreational) from the Dutch cadastre (BAG, 2019). The Shannon diversity index was used to assess the degree of land-use mix. Values close to zero indicate low diversity, higher ones indicate pronounced diversity. 

Residential density, namely the number of dwellings per buffer (in km^2^), was abstracted from the BAG (2019). Higher residential densities are thought to encourage walking, because destinations are brought closer together, thereby shortening trip distances [22]. 

Street connectivity refers to the directness and availability of alternative routes between home and local destinations [12]. It was calculated by dividing the number of at least three-way intersections per buffer (in km^2^). We derived intersections from street network data provided by the Environmental Systems Research Institute (2008). 

These three components of the walkability index were *z*-scored to permit their comparability and summed [30]. Finally, the index range was classified into quartiles, which is a common approach to ranking walkability indexes [36]. The first quartile represents the least walkable neighborhoods, and the upper quartiles represent the most walkable neighborhoods. 

### 2.4. Confounders

As sedentary behavior is performed by individuals, an adjustment must be made for demographic and socioeconomic characteristics [37,38,39]. Therefore, the questionnaire included questions regarding gender (male or female), age (in years), educational attainment (having at least a Bachelor’s degree, not having a Bachelor’s degree), employment status (employed, not employed), marital status (married, not married), and having a driver’s license (yes, no). Additionally, we added a socioeconomic status score (SES) developed by the Dutch Social and Cultural Planning Agency (Sociaal en Cultureel Planbureau) for the year 2017. This score took into account residents’ educational attainment, income, and labor market position, and may affect sedentary behavior independent of person-level characteristics. Higher scores refer to better socioeconomic conditions.

### 2.5. Statistical Analyses

Descriptive statistics were used to summarize the characteristics of the study population and to assess neighborhood walkability across the three buffer sizes. To examine correlations between neighborhood walkability and adults’ sedentary behavior levels, a series of multivariate regression models were fitted. The sedentary behaviors were the outcome variables, while the walkability index and the demographic and socioeconomic characteristics served as explanatory variables. The outcome variables deviated from a Gaussian distribution (Shapiro–Wilk test, *p* < 0.01). We fitted generalized linear models (GLM) [40] with a gamma distribution to take into account our non-negative and positively skewed outcome variables. To facilitate comparisons with earlier studies and allow for a possible non-linear association, the walkability indicator was divided into quartiles.

Two models were estimated per outcome variable. The first included only the walkability index, while the second model was fully adjusted for the confounders. McFadden’s *R^2^* was used to assess a model’s fit. An *R^2^* of between 0.2–0.4 represents an ‘excellent’ model fit. The significance level, for all analyses, was set at 0.05. The statistical analyses were carried out in IBM SPSS, version 25.0. 

### 2.6. Ethics

The survey was carried out voluntarily and consent was acquired. Respondents had the right to withdraw. Data were anonymous. Ethical approval (S-19222) was obtained from the Ethics Review Board of the Faculty of Beta- and Geosciences at Utrecht University.

## 3. Results

### 3.1. Descriptive Statistics

Descriptive statistics are presented in Table 2, stratified by the degree of neighborhood walkability. On average, the participants sat or lay down for 4.0 h per day looking at screens, and spent 1.0 h per day eating meals, and 3.4 h per day relaxing. This amounted to an average of 8.4 h per day of total sedentary time. Participants were predominantly female (58.5%) with a mean age of 45 years. They tended to be well-educated (59% had at least a Bachelor’s degree), employed (72.7%), and hold a driver’s license (94%). Around half of them were married (44.3%). The walkability index ranged from −3.8 to 6.4 for the 200-m buffer, −4.5 to 6.6 for the 400-m buffer, and −6.2 to 5.5 for the 800-m buffer. 

### 3.2. Regression

Table 3, Table 4, Table 5 and Table 6 depict the regression results. The first model across the tables comprised only the walkability index, in which the first (Q1) and fourth (Q4) quartiles represented the least and most walkable areas. The second model was fully adjusted. The first models showed no significant correlations between walkability and time spent looking at screens (Table 3), eating meals (Table 4), and relaxing (Table 5), and total time spent behaving sedentarily (Table 6). This result was consistent across all buffer distances. A full model adjustment did not alter these findings; walkability remained insignificant. 

The adjusted models showed that some demographic and socioeconomic variables were statistically significant. Educational attainment was significantly associated with screen time (β = 0.319, *p* < 0.01), relaxing (β = 0.343, *p* < 0.01), and total sedentary time (β = 0.277, *p* < 0.01). Participants without a Bachelor’s degree spent more time on sedentary behavior than participants with a Bachelor’s degree. Employment status was also significantly associated with screen time (β = 0.264, *p* < 0.05), relaxing (β = 0.344, *p* < 0.01), and total sedentary time (β = 0.276, *p* < 0.01). Unemployed participants had higher sedentary behavior levels. Having a driver’s license was also significant, but only for screen time (β = 0.443, *p* < 0.01) and total sedentary time (β = 0.284, *p* < 0.01). Participants without a driver’s license spent more time on sedentary behavior. 

The likelihood ratio tests showed that the first models did not significantly outperform an intercept-only model. This result is valid for all outcome variables. Only after adjusting for the demographic and socioeconomic characteristics in the second models did the models start to outperform an intercept-only model. Significant likelihood ratio test values were found for the outcome variables screen time, relaxing, and total sedentary time (*p* < 0.01). McFadden’s *R^2^* for the second models were as follows: 0.197 for screen time, 0.058 for eating meals, 0.139 for relaxing, and 0.239 for total sedentary time.

## 4. Discussion

### 4.1. Main Findings and Interpretation

Our results show that adults living in highly walkable neighborhoods did not spend significantly less time on sedentary behaviors than their counterparts in less walkable neighborhoods. This suggests, in contrast to our hypothesis, that factors such as mixed land-use, densely populated areas, and better-connected streets do not necessarily influence adults’ levels of sedentary behavior. 

Others found that adults living in highly walkable neighborhoods walk significantly more than adults in less walkable neighborhoods do, and spend more time being physically active [41]. It may be the case that adults in highly walkable neighborhoods replace their motorized trips with walking trips [25,26]; however, we did not test this at this stage, because most motorized travel does not take place in the residential environment. Earlier research also showed that adults living in highly walkable neighborhoods spend less time watching television [26,27], and that walking is an effective means to increase physical activity levels [42]. The present study showed that, when investigating several other screen-related activities that evoke sedentary behavior, the differences in screen time between adults living in low-walkable and high-walkable neighborhoods even out. In other words, neighborhood walkability may influence television viewing time, but its significant influence disappears when multiple other screen-related activities are taken into account. However, prolonged sitting is not the only mechanism of television viewing time that affects health outcomes [26]. Watching television is considered a more harmful sedentary behavior than playing computer games and using the computer for non-work-related purposes [43], because individuals tend to snack during their viewing time, and are also exposed to unhealthy food advertising [26]. Another study found a correlation in the unexpected direction for a total sedentary time measure [28], suggesting that adults living in highly walkable neighborhoods spend more time on sedentary behavior. Our findings may deviate from theirs, because only the sedentary behaviors in living environments were of interest, while [28] also included other domains of sedentary behavior. 

Another reason for diverging results may be the way that walkability was assessed [25,26,27,28]. Due to the lack of a gold standard to quantify walkability, different components are considered, as are different weightings of the components [18]. Other potential reasons for our lacking association is the way that walkability, and particularly land-use diversity, was assessed. Elsewhere, it was shown that changing the combination of land uses in the land used diversity assessment may translate into varying magnitudes of the estimated coefficients [44]. Moreover, the spatial context of a study is crucial, because walking plays a different role in different countries, and the built environment varies significantly. The previous studies were conducted in, for example, the USA [26], Australia [27], and Belgium [28], and the built environment of North American cities is hardly comparable with a Dutch urban morphology (more compact, more oriented toward walking and cycling, etc.), and could lead to lower as well as higher levels of sedentary behavior in both low-walkable and high-walkable environments. 

From an ecological perspective, our models were adjusted for person-level factors [10]. Adults who were higher educated, employed, and in possession of a driver’s license reported significantly lower levels of screen time and total sedentary time. Similar findings were reported elsewhere [4,37,38]. However, being higher educated and employed was associated with lower levels of relaxing, which had not previously been reported. Adult’s gender, age, and marital status were uncorrelated with time spent on sedentary behavior. Previous results are inconclusive in this respect [37,38]. Similar to our results, previous studies also concluded that lower socioeconomic status is associated with lower levels of sedentary behavior [45].

### 4.2. Strengths and Limitations

This study combined data on different sedentary behaviors with precise indicators describing the built environment, which led to novel insights into how neighborhood walkability correlates with sedentary behavior. Our objective walkability measure is less biased in term of an adult’s environmental perceptions, which may vary from person to person. Moreover, our GIS-based buffers resulted in a refined walkability assessment while circumventing some limitations of administrative units [46]. A major strength of this study is that it was not limited to one specific sedentary behavior or one total sedentary time component, as it was the case in earlier work [24,25]. Our study provides a more comprehensive assessment. Another strength was considering only a single urban area, which limited the need to control for large-scale variations in the built environmental correlates. Moreover, the walkability index was calculated for individualized neighborhoods based on street network buffers instead of postcode areas or census tracts, as done previously [26,28]. Finally, the sensitivity analysis with different buffer radii made it possible to investigate the extent to which the influence of neighborhood walkability fluctuated across scales.

However, several limitations need to be considered when interpreting our findings. First, despite being a step ahead of previous studies that used administrative boundaries, the road network buffers may not represent adults’ actual daily moving and activity patterns accurately, because people spend a significant part of their daily lives outside their residential homes. Second, although guided by earlier studies on walkability [18], the built environment was operationalized through three characteristics. We cannot exclude that other factors, such as local service provision, social interaction, littering, vandalism, and pavement quality [47,48], may influence people’s walking behavior. Third, domain-specific sedentary behaviors were self-reported. Retrieved data may be subject to measurement errors. The questionnaire was found to slightly overestimate sedentary time [29]. Moreover, we considered home-specific sedentary behavior in relation to residential neighborhood walkability. Due to a lack of data, we were not able to assess sedentary behavior at people’s workplace or during travel [7]. Since sedentary behavior levels in these other domains also affect the residential setting [10], our results should be treated with caution. Fourth, the sample size was small, but was in line with the samples used in other studies [49]. Fifth, despite adjusting for the most important confounders, it is likely that some confounders (e.g., the presence of young children) were missed. Lastly, statements about causality cannot be made due to the cross-sectional research design.

## 5. Conclusions

This study investigated the extent to which objectively measured neighborhood walkability correlates with a variety of sedentary behaviors in adults. Our results suggested that walkability is not significantly related to sedentary behaviors that occur frequently in the living environment. We also found that adults who were higher educated, employed, and had a driver’s license did show significantly lower levels of sedentary behavior. These person-level characteristics may prove more useful than the built environment for developing interventions aimed at decreasing sedentary time in adults’ living environment. 

## Figures and Tables

**Table 1:** Sedentary behaviors covered in our survey.

Class	Sedentary Behavior
*Screen time*	On average, how long did you spend sitting or lying down in the following activities per day: Watching programs, series, films and/or videos on a broadcasting medium (1), playing computer games (2), and using a computer, apart from work (3)?
*Eating meals*	On average, how long did you sit for each of these meals per day: breakfast (4), lunch (5), dinner (6)?
*Relaxing*	On average, how long did you spend sitting or lying down in the following activities per day: reading (7), pursuing hobbies (8), listening to music (9), and socializing with others (10)
*Total sedentary time*	A combination of all individual behaviors

**Table 2:** Descriptive statistics stratified by neighborhood walkability across three buffer sizes.

		Neighborhood Walkability (200 m)		Neighborhood Walkability (400 m)		Neighborhood Walkability (800 m)	
	Total (*n* = 183)	Low ^A^ (*n* = 46)	High ^B^ (*n* = 46)	Low ^A^ (*n* = 46)	High ^B^ (*n* = 46)	Low ^A^ (*n* = 46)	High ^B^ (*n* = 46)
*Sed. behavior (h): mean (SD)*							
Screen time	4.0 (2.6)	4.0 (2.4)	4.0 (2.8)	3.8 (2.3)	4.4 (3.1)	4.2 (2.6)	4.0 (2.9)
Eating	1.0 (0.5)	1.0 (0.5)	0.9 (0.5)	1.0 (0.5)	1.0 (0.5)	1.1 (0.4)	1.0 (0.7)
Relaxing	3.4 (2.8)	3.2 (1.8)	3.2 (2.4)	3.0 (1.9)	3.7 (3.5)	3.0 (1.7)	3.4 (2.8)
Total SB	8.4 (4.3)	8.2 (3.7)	8.2 (4.1)	7.9 (3.4)	9.1 (4.7)	8.3 (3.8)	8.4 (4.6)
*Gender (%)*							
Male	41.0	47.8	33.3	32.6	35.6	37.0	35.6
Female	58.5	52.2	66.7	67.4	64.4	63.0	64.4
*Age: mean (SD)*	45.2 (16.5)	45.0 (16.3)	40.0 (16.4)	49.4 (14.7)	40.5 (15.8)	49.3 (16.4)	41.0 (16.7)
*Educational attainment (%)*							
At least a Bachelor’s degree	59.0	56.5	58.7	58.7	56.5	54.3	54.3
No Bachelor’s degree	41.0	43.5	41.3	41.3	43.5	45.7	45.7
*Employment status (%)*							
Employed	72.7	78.3	71.7	73.9	73.9	71.7	69.6
Unemployed	27.3	21.7	28.3	26.1	26.1	28.3	30.4
*Marital status (%)*							
Married	44.3	58.7	30.4	54.3	30.4	58.7	37.0
Not married	55.7	41.3	69.9	45.7	69.6	41.3	63.0
*Driver’s license (%)*							
Has a license	94.0	93.5	97.8	100.0	89.1	97.8	91.3
Does not have a license	6.0	6.5	2.2	0.0	10.9	2.2	8.7
*SES score: mean (SD)*	0.2 (1.1)	0.2 (1.0)	0.1 (1.1)	0.5 (0.7)	−0.1 (1.2)	0.7 (0.7)	−0.1 (1.1)

**^A^** Low refers to the first quartile (i.e., the least walkable neighborhoods). **^B^** High refers to the fourth quartile (i.e., the most walkable neighborhoods).

**Table 3:** Results of the gamma generalized linear models (GLM) for time spent looking at screens.

	Neighborhood Walkability 200 m	Neighborhood Walkability 400 m	Neighborhood Walkability 800 m
Models	β (95% CI)	β (95% CI)	β (95% CI)
*Model 1*			
Intercept	1.375 (1.2, 1.6) **	1.348 (1.2, 1.5) **	1.428 (1.3, 1.6) **
WI Quartile 2 (ref: Q1)	0.052 (−0.2, 0.3)	0.129 (−0.1, 0.4)	0.035 (−0.2, 0.3)
WI Quartile 3 (ref: Q1)	−0.014 (−0.3, 0.2)	−0.125 (−0.4, 0.1)	−0.161 (−0.4, 0.1)
WI Quartile 4 (ref: Q1)	0.023 (−0.2, 0.3)	0.142 (−0.1, 0.4)	−0.038 (−0.3, 0.2)
χ2 Likelihood ratio test	0.300	5.661	2.604
Deviance	73.204	71.211	72.341
McFadden’s *R^2^* *Model 2*	0.002	0.029	0.013
Intercept	0.952 (0.6, 1.3) **	0.955 (0.6, 1.4) **	1.047 (0.6, 1.4) **
WI Quartile 2 (ref: Q1)	−0.026 (−0.3, 0.2)	0.046 (−0.2, 0.3)	0.038 (−0.2, 0.3)
WI Quartile 3 (ref: Q1)	−0.006 (−0.2, 0.2)	-0.124 (−0.4, 0.1)	−0.133 (−0.4, 0.1)
WI Quartile 4 (ref: Q1)	0.050 (−0.2, 0.3)	0.125 (−0.1, 0.4) *	−0.077 (−0.3, 0.2)
Gender (ref: male)	0.077 (−0.1, 0.2)	0.077 (−0.1, 0.2)	0.068 (−0.1, 0.2)
Age	0.002 (−0.0, 0.0)	0.002 (−0.0, 0.0)	0.001 (−0.0, 0.0)
Educ. att. (ref: has Bachelor’s deg.)	0.326 (0.2, 0.5) **	0.318 (0.1, 0.5) **	0.312 (0.1, 0.5) **
Employment (ref: employed)	0.258 (0.1, 0.5) *	0.270 (0.1, 0.5) *	0.264 (0.1, 0.5) **
Marital status (ref: married)	0.046 (−0.1, 0.2)	0.038 (−0.1, 0.2)	0.052 (−0.1, 0.2)
Driver’s license (ref: has a license)	0.466 (0.1, 0.8) **	0.414 (0.1, 0.8) **	0.449 (0.1, 0.8) **
SES scores	0.018 (−0.1, 0.1)	0.019 (−0.1, 0.1)	−0.006 (−0.1, 0.1)
χ2 Likelihood ratio test	37.409 **	41.578 **	39.128 **
Deviance	59.458	58.176	58.926
McFadden’s *R^2^*	0.189	0.207	0.196

Significance codes: * *p* < 0.05; ** *p* < 0.01. WI = walkability index. CI = confidence interval.

**Table 4:** Results of the gamma GLM for time spent eating meals.

	Neighborhood Walkability 200 m	Neighborhood Walkability 400 m	Neighborhood Walkability 800 m
Models	β (95% CI)	β (95% CI)	β (95% CI)
*Model 1*			
Intercept	0.034 (−0.1, 0.2)	−0.006 (−0.1, 0.1)	0.060 (−0.1, 0.2)
WI Quartile 2 (ref: Q1)	0.097 (−0.1, 0.3)	0.061 (−0.1, 0.3)	−0.023 (−0.2, 0.2)
WI Quartile 3 (ref: Q1)	0.018 (−0.2, 0.2)	0.126 (−0.1, 0.3)	−0.012 (−0.2, 0.2)
WI Quartile 4 (ref: Q1)	−0.082 (−0.3 0.1)	0.013 (−0.2, 0.2)	−0.023 (−0.2, 0.2)
χ2 Likelihood ratio test	3.069	1.857	0.067
Deviance	45.008	45.297	45.727
McFadden’s *R^2^* *Model 2*	0.016	0.010	0.000
Intercept	0.087 (−0.3, 0.4)	−0.002 (−0.4, 0.4)	0.083 (−0.3, 0.4)
WI Quartile 2 (ref: Q1)	0.098 (−0.1, 0.3)	0.049 (−0.2, 0.3)	−0.044 (−0.2, 0.2)
WI Quartile 3 (ref: Q1)	0.051 (−0.2, 0.3)	0.132 (−0.1, 0.3)	0.004 (−0.2, 0.2)
WI Quartile 4 (ref: Q1)	−0.027 (−0.2, 0.2)	0.039 (−0.2, 0.2)	−0.006 (−0.2, 0.2)
Gender (ref: male)	0.032 (−0.1, 0.2)	0.037 (−0.1, 0.2)	0.031 (−0.1, 0.2)
Age	−0.001 (−0.0, 0.0)	0.000 (−0.0, 0.0)	0.000 (−0.0, 0.0)
Educ. att. (ref: has Bachelor’s deg.)	−0.018 (−0.2, 0.1)	−0.020 (−0.2, 0.1)	−0.028 (−0.2, 0.1)
Employment (ref: employed)	0.087 (−0.1, 0.3)	0.090 (−0.1, 0.3)	0.096 (−0.1, 0.3)
Marital status (ref: married)	−0.132 (−0.3, 0.0)	−0.119 (−0.3, 0.0)	−0.117 (−0.3, 0.0)
Driver’s license (ref: has a license)	0.278 (−0.0, 0.6)	0.281 (−0.0, 0.6)	0.298 (−0.0, 0.6)
SES scores	0.013 (−0.1, 0.1)	0.019 (−0.1, 0.1)	0.019 (−0.1, 0.1)
χ2 Likelihood ratio test	9.455	9.621	8.113
Deviance	43.011	42.973	43.320
McFadden’s *R^2^*	0.060	0.061	0.053

Significance codes: * *p* < 0.05; ** *p* < 0.01. WI = walkability index. CI = confidence interval.

**Table 5:** Results of the gamma GLM for time spent relaxing.

	Neighborhood Walkability 200 m	Neighborhood Walkability 400 m	Neighborhood Walkability 800 m
Models	β (95% CI)	β (95% CI)	β (95% CI)
*Model 1*			
Intercept	1.161 (1.0, 1.4) **	1.124 (0.9, 1.3) **	1.128 (0.9, 1.3) **
WI Quartile 2 (ref: Q1)	0.238 (−0.1, 0.5)	0.212 (−0.1, 0.5)	0.195 (−0.1, 0.5)
WI Quartile 3 (ref: Q1)	−0.003 (−0.3, 0.3)	−0.024 (−0.3, 0.3)	0.095 (−0.2, 0.4)
WI Quartile 4 (ref: Q1)	0.040 (−0.3, 0.3)	0.228 (−0.1, 0.5)	0.127 (−0.2, 0.4)
χ2 Likelihood ratio test	3.323	4.437	1.604
Deviance	105.535	104.993	106.470
McFadden’s *R^2^* *Model 2*	0.017	0.022	0.008
Intercept	1.048 (0.6, 1.5) **	0.993 (0.7, 1.7) **	0.939 (0.4, 1.4) **
WI Quartile 2 (ref: Q1)	0.152 (−0.1, 0.5)	0.122 (−0.2, 0.4)	0.197 (−0.1, 0.5)
WI Quartile 3 (ref: Q1)	−0.052 (−0.3, 0.2)	−0.048 (−0.3, 0.2)	0.081 (−0.2, 0.4)
WI Quartile 4 (ref: Q1)	0.031 (−0.3, 0.3)	0.199 (−0.1, 0.5)	0.134 (-0.2, 0.4)
Gender (ref: male)	−0.074 (−0.3, 0.1)	−0.087 (−0.3, 0.1)	−0.097 (−0.3, 0.1)
Age	−0.002 (−0.0, 0.0)	−0.001 (−0.0, 0.0)	−0.001 (−0.0, 0.0)
Educ. att. (ref: has Bachelor’s deg.)	0.344 (0.1, 0.6) **	0.330 (0.1, 0.6) **	0.355 (0.1, 0.6) **
Employment (ref: employed)	0.324 (0.1, 0.6) *	0.358 (0.1, 0.6) **	0.351 (0.1, 0.6) **
Marital status (ref: married)	0.056 (−0.2, 0.3)	0.044 (−0.2, 0.3)	0.051 (−0.2, 0.3)
Driver’s license (ref: has a license)	0.066 (−0.4, 0.5)	−0.026 (−0.5, 0.4)	0.019 (−0.4, 0.5)
SES scores	−0.030 (−0.1, 0.1)	−0.017 (−0.1, 0.1)	−0.019 (−0.1, 0.1)
χ2 Likelihood ratio test	24.019 **	25.345 **	23.990 **
Deviance	92.644	92.006	92.658
McFadden’s *R^2^*	0.137	0.143	0.137

Significance codes: * *p* < 0.05; ** *p* < 0.01. WI = walkability index. CI = confidence interval.

**Table 6:** Results of the gamma GLM for total sedentary time.

	Neighborhood Walkability 200 m	Neighborhood Walkability 400 m	Neighborhood Walkability 800 m
Models	β (95% CI)	β (95% CI)	β (95% CI)
*Model 1*			
Intercept	2.102 (2.0, 2.2) **	2.061 (1.9, 2.2) **	2.111 (2.0, 2.3) **
WI Quartile 2 (ref: Q1) A	0.115 (−0.1, 0.3)	0.153 (−0.0, 0.4)	0.090 (−0.1, 0.3)
WI Quartile 3 (ref: Q1)	−0.005 (−0.2, 0.2)	−0.042 (−0.2, 0.2)	−0.032 (−0.2, 0.2)
WI Quartile 4 (ref: Q1)	−0.003 (−0.2, 0.2)	0.150 (−0.0, 0.3)	0.015 (−0.2, 0.2)
χ2 Likelihood ratio test	2.080	5.961	1.555
Deviance	44.682	43.778	44.805
McFadden’s *R^2^* *Model 2*	0.011	0.031	0.008
Intercept	1.859 (1.6, 2.2) **	1.812 (1.6, 2.2) **	1.854 (1.6, 2.1) **
WI Quartile 2 (ref: Q1)	0.048 (−0.1, 0.2)	0.084 (−0.1, 0.3)	0.089 (−0.1, 0.3)
WI Quartile 3 (ref: Q1)	−0.018 (−0.2, 0.2)	−0.042 (−0.2, 0.1)	−0.023 (−0.2, 0.2)
WI Quartile 4 (ref: Q1)	0.009 (−0.2, 0.2)	0.132 (−0.1, 0.3)	0.003 (−0.2, 0.2)
Gender (ref: male)	0.011 (−0.1, 0.1)	0.009 (−0.1, 0.1)	−0.001 (−0.1, 0.1)
Age	0.001 (−0.0, 0.0)	0.001 (−0.0, 0.0)	0.001 (−0.0, 0.0)
Educ. att. (ref: has Bachelor’s deg.)	0.278 (0.1, 0.4) **	0.273 (0.1, 0.4) **	0.279 (0.1, 0.4) **
Employment (ref: employed)	0.269 (0.1, 0.4) **	0.279 (0.1, 0.4) **	0.281 (0.1, 0.4) **
Marital status (ref: married)	0.009 (−0.1, 0.2)	0.001 (−0.1, 0.1)	0.008 (−0.1, 0.2)
Driver’s license (ref: has a license)	0.303 (0.0, 0.6) *	0.259 (−0.0, 0.5)	0.290 (0.0, 0.6)*
SES scores	−0.001 (−0.1, 0.1)	0.006 (−0.1, 0.1)	−0.005 (−0.1, 0.1)
χ2 Likelihood ratio test	44.098 **	47.960 **	45.306 **
Deviance	34.691	33.984	34.468
McFadden’s *R^2^*	0.232	0.248	0.237

Significance codes: * *p* < 0.05; ** *p* < 0.01. WI = walkability index. CI = confidence interval.
